# Hashimoto’s disease - from theory to practice

**DOI:** 10.1186/1756-6614-6-S2-A60

**Published:** 2013-04-05

**Authors:** Anhelli Syrenicz

**Affiliations:** 1 Department of Endocrinology, Metabolic Diseases and Internal Diseases, Pomeranian Medical University, Szczecin, Poland

## 

Hashimoto’s thyroiditis is the most prevalent autoimmune thyroid gland disease. It is now over a century since the first description of the disease by the Japanese doctor Hakaru Hashimoto in 1912, yet the etiopathogenesis of the disease is still discussed. At present it is thought that Hashimoto’s disease is provoked in genetically susceptible individuals by both environmental and endogenous triggers. Genetic predisposition to development of the autoimmune thyroid diseases was established on the basis of the epidemiologic studies indicating increased prevalence of such diseases in some families, especially in twins. According to current knowledge appearance of Hashimoto’s disease in Caucasians is associated with some gene alleles: human leukocyte antigens (HLAs), mainly class II HLA DR3 and DR 5, cytotoxic T lymphocyte antigen-4 (CTLA-4), protein tyrosine phosphatase nonreceptor – type 22 (PTPN22), thyroglobulin (Tg), vitamin D receptor (VDR) and cytokines. Among environmental factors triggering the thyroid autoimmunity the following should be mentioned: excessive iodine intake, treatment with certain drugs (interferon α, IL-2, lithium, amiodarone), infections, mainly viral and exposure to many chemicals such as polyaromatic hydrocarbons and phenyls. Female sex, rebound phenomenon in postpartum period and fetal microchimerism are essential endogenous factors in the etiopathogenesis of Hashimoto’s disease. Above mentioned factors are responsible for the development of autoimmune response in thyroid gland. It leads to increased antigen presentation by antigen presenting cells (APC), inappropriate presentation of HLA antigens class II by thyroid follicular cells and reduced immune tolerance. Developing autoimmune process, predominantly Th1-type, is responsible for the increased production of TNF-α, IFN-γ and IL-1 cytokines. Destruction of thyroid tissue with subsequent development of fibrous tissue is mediated by apoptosis process, CD8+ cytotoxicity, change of cell junctions and complement activation. On clinical examination Hashimoto’s disease may present as classical, atrophic, focal or juvenile form. Additionally, there are two variants of Hashimoto’s disease: silent, painless thyroiditis and postpartum thyroiditis. Natural course of Hashimoto’s disease leads to hypothyroid state. High antiperoxidase and antithyroglobulin antibodies concentrations and hypoechogenic structure of thyroid gland on ultrasonographic examination confirm the diagnosis of Hashimoto’s disease. Fine-needle aspiration biopsy is rarely needed to confirm the diagnosis. The treatment of Hashimoto’s disease includes an administration of substitutive doses of levothyroxine, but the time of treatment beginning is still the matter of discussion.

